# Variation in Uterine and Serum Proteins During the Reproductive Cycle in Dairy Cows Following Low and High Dietary Protein Intake

**DOI:** 10.1002/vms3.70631

**Published:** 2025-10-04

**Authors:** Akbar Pirestani, Elmira Ziya Motalebipour

**Affiliations:** ^1^ Department of Animal Science Institute of Agriculture Water Food and Nutraceuticals Isf. C. Islamic Azad University Isfahan Iran; ^2^ Department of Agronomy and Plant Breeding Institute of Agriculture Water Food and Nutraceuticals Isf. C. Islamic Azad University Isfahan Iran; ^3^ Transgenesis Center of Excellence Isf. C. Islamic Azad University Isfahan Iran; ^4^ Medicinal Plants Research Center Isf. C. Islamic Azad University Isfahan Iran

**Keywords:** blood serum protein, cow's reproductive cycle, dietary protein, uterine fluid

## Abstract

**Objectives:**

This study aimed to investigate the correlation between dietary protein levels and the concentrations of various proteins in blood serum and uterine fluid during different stages of the reproductive cycle in dairy cows.

**Materials and Methods:**

Forty Holstein cows, all 45 days postpartum, were randomly assigned to two groups: one receiving a diet containing 16% protein, and the other 19% protein. After a 14‐day adaptation period, oestrus synchronization was performed using the Heatsynch protocol. Cows were fed total mixed rations (TMR) three times daily, adjusted based on weight and age. Blood and uterine fluid samples were collected at different stages of the reproductive cycle and analysed for total protein, albumin and globulin fractions (alpha‐1, alpha‐2, beta‐1, beta‐2 and gamma globulin).

**Results:**

Cows receiving the 19% protein diet exhibited significantly higher uterine fluid total protein levels across all cycle phases (*p* < 0.05), and increased uterine albumin during proestrus, oestrus and metestrus. In serum, alpha‐1‐globulin was higher in the 19% group during oestrus and metestrus, whereas alpha‐2‐, beta‐1‐, beta‐2‐ and gamma‐globulins were generally higher in the 16% group at specific phases (*p *< 0.05). Pearson correlations revealed stage‐specific relationships between serum and uterine proteins, with alpha‐2‐globulin and gamma‐globulin showing opposing correlation patterns during dioestrus. No significant differences were observed in fertility or pregnancy rates between treatments.

**Conclusion:**

Higher dietary protein levels appear to influence reproductive parameters in dairy cows by altering protein profiles in blood and uterine fluid. These findings highlight the role of dietary management in optimizing reproductive health and performance.

## Graphical abstract

This study investigates the effect of different dietary protein levels (16% and 19%) on serum and uterine fluid protein concentrations in dairy cows at various stages of the reproductive cycle. The results showed that the diet with 19% protein induced significant changes in protein profiles and may enhance reproductive health and performance. The importance of nutritional management in optimizing reproduction is highlighted.

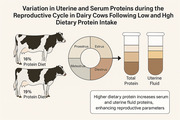

## 1 Introduction

Ensuring food security through efficient livestock production is a central goal in both developed and developing countries. Among livestock sectors, dairy cattle play a critical role in producing milk and other dairy products. However, due to limitations in production resources, enhancing reproductive efficiency is key to maximizing productivity (Abdikerimova and Moldabekov 2021). Fertility is a major determinant of profitability in dairy herds. One commonly adopted strategy to boost milk yield, particularly during early lactation, is increasing dietary protein intake. While this can improve milk production, high‐protein diets have also been associated with reduced reproductive performance, highlighting a complex interaction between dietary protein and fertility (De Vries and Marcondes 2020).

A major concern with excessive dietary protein is the elevated concentration of blood urea nitrogen (BUN) in postpartum cows, which leads to higher levels of urea and ammonia in the reproductive tract. This biochemical environment can reduce uterine pH and adversely affect oocyte and embryo quality (Boakari et al. 2021). Therefore, balancing protein intake is crucial not only for milk production but also for successful conception and pregnancy maintenance.

In ruminants, protein requirements are met through both true protein and non‐protein nitrogen (NPN) sources. Excess dietary nitrogen, whether from true protein or NPN, is converted into urea in the liver and distributed throughout body fluids, including blood and milk (Panday 2010). Urea levels in these fluids can reflect protein metabolism and may influence reproductive tract conditions.

Serum proteins, which include albumin (Alb) and various globulins (Glb) (Kaneko et al. 1997; Keay and Doxey 1981), are vital biomarkers of metabolic and immune status. Similarly, uterine proteins play an essential role in embryo development and pregnancy establishment by regulating the uterine environment and embryo–maternal communication. Altered protein composition in uterine fluid may indicate impaired reproductive function.

The biological value of dietary protein is influenced by its digestibility and amino acid composition, as well as the cow's energy balance. High crude protein intake, particularly rumen‐degradable protein, may impair fertility by increasing metabolic stress and reducing plasma progesterone levels (Beam and Butler 1997; Stallings et al. 1991).

Recent studies have emphasized the importance of uterine proteins in the fertilization process and early embryonic development. Changes in metabolic substrates such as glucose, amino acids and short‐chain fatty acids can alter uterine secretions and compromise embryo viability (Groebner et al. 2011; Leroy et al. 2014).

Despite growing evidence linking dietary protein with reproductive outcomes, the precise relationship between protein intake, serum protein profiles and uterine protein composition across different reproductive stages remains unclear. This study aims to address this gap by evaluating how dietary protein levels influence the protein content of blood serum and uterine fluid during the reproductive cycle in dairy cows.

## 2 Material and Methods

### 2.1 Animals and Housing

A total of 40 multiparous Holstein dairy cows, approximately 45 days post‐calving and with similar body condition scores and the same number of parturitions, were enrolled in this experiment. The study was conducted in spring 2020 at Alian Livestock Farm, located along the Tehran‐Kashan highway in Isfahan province. Cows were housed in open‐sided free‐stall barns with straw bedding and had free access to water and shade.

### 2.2 Experimental Design, Diets and Feeding

Cows were randomly assigned to two dietary treatment groups (*n* = 20 cows per group) based on parity, age and milk yield. The experimental treatments consisted of total mixed rations (TMR) containing either 16% or 19% crude protein. The chosen levels reflect common dietary formulations for high‐producing dairy cows, allowing investigation into the potential reproductive effects within this practical range. However, future studies may consider including wider protein extremes for broader comparison.

Diets were formulated using NRC (2007) standards and AminoCow software to meet the cows’ nutritional requirements. Rations were offered three times per day, and feed intake was monitored throughout the trial. Detailed ingredients and chemical composition of the diets are presented in Table 1. The feeding and sampling period lasted for one full oestrous cycle (∼21 days), following a 14‐day dietary adaptation period.

**TABLE 1:** Composition of experimental diets fed for the 16% and 19% dietary crude protein (CP) level.

No.	Combination	Amount (16% CP diet)	Amount (19% CP diet)
1	Concentrate (kg per day)	8.4	8.5
2	Hay (kg per day)	2.7	3.6
3	Corn silage (kg per day)	5.6	6.6
4	soybean meal (kg per day)	1.5	3.2
5	Crude protein (%DM)	16	19
6	Calcium (%DM)	0.8	0.8
7	Phosphorus (%DM)	0.5	0.4
8	Net energy (Mcal/k)	1.67	1.72

### 2.3 Measured Indicators

#### 2.3.1 Oestrus Synchronization and Sampling

After the adaptation period, all cows underwent oestrus synchronization using the Heatsynch protocol (Pancarci et al. 2002; Yusuf et al. 2010). The synchronization regimen involved intramuscular injection of GnRH on Day 0, PGF2α on Day 7 and estradiol cypionate (ECP) 32 h later.

Blood samples (10 mL) were collected from the coccygeal vein at two time points: once before synchronization and again on the day of oestrus detection. Uterine fluid was aspirated using a sterile Foley catheter during each phase of the oestrous cycle: proestrus, oestrus, metestrus and dioestrus. All samples were transported on ice and stored at −20°C until analysis.

#### 2.3.2 Biochemical Analyses

Blood serum and uterine fluid samples were analysed for total protein (TP), Alb and Glb concentrations using commercial biochemical kits (Pars Azmoon Co., Tehran, Iran) and a spectrophotometric method. TP was measured based on a colourimetric assay in which proteins form a blue‐coloured complex with copper ions in an alkaline medium, with colour intensity proportional to protein concentration. Serum Alb, a non‐glycosylated single‐chain protein, was determined by its reaction with bromocresol green at acidic pH, producing a green‐blue complex measured spectrophotometrically. Glb concentration was calculated by subtracting Alb from TP.

Furthermore, the separation and quantification of Glb fractions (alpha‐1, alpha‐2, beta‐1, beta‐2 and gamma‐Glbs) were performed using an electrophoresis system. In this method, serum samples are applied to a solid medium—typically agarose or cellulose acetate—and subjected to an electric field. Proteins migrate through the medium at different rates based on their molecular size, charge and shape, resulting in the formation of distinct bands. After staining with protein‐specific dyes, the bands are visualized and quantified using image analysis software.

#### 2.3.3 Reproductive Performance

The reproductive parameters measured in this study included the service/pregnancy rate, %heat detection rate (HDR), %conception rate (CR) and %pregnancy rate. Pregnancy diagnosis was performed using the rectal test method on the 30th day after insemination with an Emperor 830 vet portable ultrasound.

$$\mathrm{Service}/\mathrm{pregnancy}\;\mathrm{rate}\;=\frac{\#\mathrm{Total}\;\mathrm{cows}\;\mathrm{AI}\;}{\#\mathrm{Cows}\;\mathrm{that}\;\mathrm{confirmed}\;\mathrm{pregnancy}\;}$$

$$\%\mathrm{HDR}=\frac{\mathrm{Heats}\;\mathrm{observed}}{\left(\mathrm{Total}\;\mathrm{cow}\;\mathrm{days}\;\mathrm{in}\;\mathrm{period}+21\right)}\;\times 100$$

$$\%\mathrm{Conception}\;\mathrm{rate}\;\;\left(\mathrm{CR}\right)=\frac{\#\mathrm{Cows}\;\mathrm{that}\;\mathrm{confirmed}\;\mathrm{pregnancy}}{\#\mathrm{Total}\;\mathrm{cows}\;\mathrm{AI}\;}\times 100$$

%Pregnancyrate=CR×HDR

### 2.4 Data Analysis

All data were analysed using a completely randomized design in SAS software (SAS Institute 2004). The General Linear Model (GLM) procedure was used to determine the effects of dietary treatments. Least Squares Means (LSMeans) were compared, and differences were considered statistically significant at *p* < 0.05.

## 3 Result

### 3.1 Effects of Dietary Protein on Serum and Uterine Protein Fractions During the Oestrous Cycle

**FIGURE 1:**
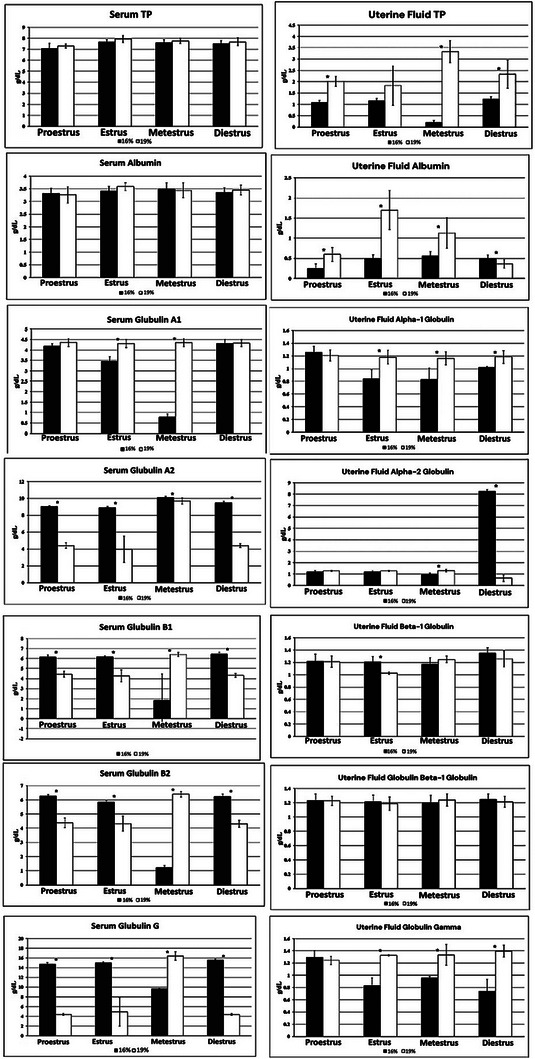
The comparison of average protein in the uterine and serum of dairy cows fed with different protein treatments across various stages of the oestrous cycle. '*': coefficients that are significant at the level of 0.05, 0.01 and 0.001, respectively.

The study evaluated the influence of two dietary protein levels (16% and 19%) on concentrations of TP, Alb and Glb fractions (alpha‐1, alpha‐2, beta‐1, beta‐2 and gamma Glb) in blood serum and uterine fluid of dairy cows during the oestrous cycle. Measurements were taken at four distinct phases: proestrus, oestrus, metaestrus and dioestrus. The data are presented in Figure 1.

In blood serum, cows fed the 19% protein diet showed numerically higher TP levels than the 16% group, though no significant differences were observed across any oestrous stage (*p* > 0.05). In uterine fluid, TP levels were significantly higher in cows receiving the 19% protein diet across all stages of the cycle (*p* < 0.05).

For Alb, there were no significant differences between treatment groups in blood serum across the oestrous cycle (*p* > 0.05). In contrast, in uterine fluid, Alb concentrations were significantly higher in the 19% group during the oestrus, metaestrus and proestrus phases (*p* < 0.05). During dioestrus, the 16% group exhibited significantly higher Alb levels than the 19% group (*p* < 0.05).

Regarding alpha‐1 Glb, blood serum levels were significantly higher in the 19% group during oestrus and metaestrus (*p* < 0.05). In uterine fluid, this group also showed significantly elevated alpha‐1 Glb concentrations during oestrus, metaestrus and dioestrus (*p* < 0.05).

Alpha‐2 Glb levels in blood serum were significantly higher in the 16% protein group during all four phases of the oestrous cycle (*p* < 0.05). In uterine fluid, the 19% group had significantly higher levels during metaestrus, while during dioestrus, the 16% group showed significantly greater concentrations (*p* < 0.05). No other significant differences were noted.

Beta‐1 Glb concentrations in blood serum were significantly greater in the 16% protein group during proestrus, oestrus and dioestrus (*p* < 0.05). Conversely, during metaestrus, the 19% group exhibited significantly higher levels (*p* < 0.05). In uterine fluid, a difference was observed only during oestrus, where the 16% group showed higher levels, although not statistically significant (*p* > 0.05).

For beta‐2 Glb, cows on the 16% diet had significantly higher blood serum levels during proestrus, oestrus and dioestrus stages (*p* < 0.05). In contrast, during metaestrus, the 19% group exhibited significantly higher concentrations (*p* < 0.05). In uterine fluid, no significant differences between treatment groups were detected at any stage of the cycle (*p* > 0.05).

Gamma‐Glb levels in blood serum were significantly higher in the 16% protein group during proestrus, oestrus and dioestrus (*p* < 0.05), while during metaestrus, the 19% group showed significantly higher values (*p* < 0.05). In uterine fluid, the 19% protein group exhibited significantly higher gamma Glb levels during oestrus, metaestrus and dioestrus phases compared to the 16% group (*p* < 0.05).

### 3.2 Correlation of Serum and Uterine Fluid Parameters

Pearson correlation analysis revealed significant variations in protein interactions between blood and uterine fluid across different stages of the reproductive cycle in dairy cows. In proestrus, alpha Glb 2 showed a significant negative correlation with other proteins (*r *= −0.503, *p* < 0.001), while correlations among other proteins were generally weak to moderate. During oestrus, beta Glb 1 exhibited a strong negative correlation (*r* = −0.715, *p *< 0.001), and gamma Glb showed a strong positive correlation with other proteins (*r* = 0.837, *p* < 0.001). In metestrus, alpha Glb 1 had a strong positive correlation (*r *= 0.75, *p *< 0.001), whereas alpha Glb 2 was negatively correlated (*r* = −0.515, *p* < 0.001). Moderate positive correlations were also noted in TP and beta Glb 1 (Table 2).

**TABLE 2:** Pearson's correlation coefficients between proteins measured in blood and uterine fluid in different stages of the reproductive cycle of dairy cows.

No.	Parameter	Total protein	Albumin	Alpha globulin 1	Alpha globulin 2	Beta globulin 1	Beta globulin 2	Gamma globulin
1	Proestrus	0.283	−0.085	−0.085	−0.503^***^	0.033	0.012	0.243
2	Oestrus	−0.230	−0.347^*^	−0.635^***^	0.185	−0.715^***^	−0.031	0.837^***^
3	Metestrus	0.352^*^	0.031	0.750^***^	−0.515^***^	0.389^*^	0.194	0.839^***^
4	Dioestrus	0.222	−0.068	−0.005	0.997^***^	0.430^**^	0.215	−0.913^***^

*, ** and ***: coefficients that are significant at the level of 0.05, 0.01 and 0.001, respectively.

In dioestrus, alpha Glb 2 demonstrated an extremely strong positive correlation (*r* = 0.997, *p* < 0.001), while gamma Glb had a strong negative correlation (*r* = −0.913, *p* < 0.001). Beta Glb 1 showed a moderate positive correlation (*r* = 0.43, *p *< 0.01).

### 3.3 Number of Inseminations, Fertility Rate and Pregnancy

Table 3 presents the comparison of average reproductive indices in dairy cows subjected to different dietary treatments throughout the experimental periods. Analysis of the effects of varying dietary protein levels on the number of inseminations, fertility rate and pregnancy outcomes revealed no statistically significant differences (*p* > 0.05). Although cows fed a 16% dietary protein diet required fewer inseminations per pregnancy compared to those receiving a 19% protein diet, the difference was not statistically significant. Similarly, while the fertility and pregnancy rates appeared higher in the 19% protein group, these differences were also not statistically significant between the two dietary treatments. Comparison of the means between the 16% and 19% groups for the variables of number of inseminations and pregnancy showed no significant difference between the two groups for these means.

**TABLE 3:** Comparison of the means of experimental groups in reproductive indices.

No.	Treatment group	Number of inseminations per pregnancy	Fertility rate	Pregnancy rate
1	Treatment group by 16% protein diet	2.9	85%	74%
2	Treatment group by 19% protein diet	3.05	90%	78%

## 4 Discussion

### 4.1 The Impact of Dietary Protein Levels on Serum and Uterine Protein Fractions Across the Oestrous Cycle in Dairy Cows

This study examined the influence of two dietary protein levels (16% and 19%) on TP, Alb and Glb concentrations in the blood serum and uterine fluid of dairy cows during the four phases of the oestrous cycle: proestrus, oestrus, metestrus and dioestrus. The results indicated that a 19% dietary protein level significantly increased serum TP concentrations, especially during the metestrus phase, compared to the 16% protein group. In contrast, no significant differences were observed during oestrus. Uterine fluid protein concentrations were also significantly affected by dietary protein, with the highest variations again occurring in the metestrus phase.

The role of nutrition, particularly protein intake, is fundamental to reproductive performance in ruminants, especially under conditions of nutritional stress (Howard 1987). The findings of this study support previous research indicating that increased dietary protein intake can enhance serum protein levels. However, some non‐significant differences in this study may be due to individual variations or insufficient contrast between dietary groups. Notably, excessive dietary protein (> 17%–20%) has been associated with reduced fertility, prolonged open periods and increased insemination frequency (Elrod and Butler 1993). This is attributed to elevated blood ammonia and urea levels caused by rumen‐degradable protein not matched by adequate energy intake. These nitrogenous by‐products impair fertility by disrupting uterine pH, prostaglandin production and luteinizing hormone (LH) receptor binding, ultimately reducing serum progesterone levels and embryo survival (Elrod and Butler 1993; Hammon et al. 2005).

The significant changes in uterine fluid protein are likely influenced by blood serum composition and dilution effects during oestrus. Increased oestrogen levels during oestrus contribute to uterine fluid volume, which may mask concentration changes. Moreover, high dietary protein accelerates rumen fermentation and volatile fatty acid (VFA) production, increasing metabolic protein levels and activating homeostatic gene responses to maintain equilibrium (Ramos et al. 2021). Protein intake is also known to affect oocyte quality, ovulation, embryonic development and hormone regulation (Van Saun 2004; Wang et al. 2018; Hristov et al. 2019). It also influences insulin, IGF‐I, leptin and mRNA expression in the ovarian IGF system, affecting follicular response to gonadotropins (Butler 2005).

In terms of Alb, no statistically significant differences were observed in serum between the two dietary groups, although a non‐significant increase was detected in the oestrus and dioestrus phases in cows fed 19% protein. In contrast, Alb concentration in uterine fluid was significantly higher in the 19% group across all phases, with the greatest difference observed during oestrus. This may be linked to decreased uterine pH from elevated protein intake, reducing progesterone levels and affecting lipid metabolism and steroidogenesis (Law et al. 2009). Albumin plays crucial physiological roles, including maintaining colloidal osmotic pressure, transporting bioactive compounds and acting as a protein reservoir (Deb et al. 2018). Its increased presence in the uterus during key reproductive phases supports its function as a steroid hormone transporter, potentially enhancing ovarian function (Kuten Pella et al. 2022).

Moreover, studies show that low energy and protein intake in high‐yielding dairy cows are associated with lower serum Alb and reduced fertility (Cheng et al. 2015). Albumin levels also fluctuate around parturition due to increased Glb concentrations related to colostrum production (Jordan and Swanson 1979). In addition, elevated blood urea levels from high rumen‐degradable protein intake negatively impact hormonal balance and fertility, which aligns with the current study's findings (Gilmore et al. 2011).

Regarding Glbs, alpha‐1 Glb levels in serum were significantly higher in the 19% protein group during oestrus and metestrus but not in proestrus or dioestrus. Uterine fluid alpha‐1 Glb also increased significantly in the 19% group during oestrus, metestrus and dioestrus, potentially due to shifts in the Alb‐to‐Glb ratio from increased urea and decreased Alb (Ferguson and Chalupa 1989).

For alpha‐2 Glb, serum levels were significantly higher in the 16% group throughout the oestrous cycle, possibly due to better utilization of soybean meal protein by rumen microbes. In uterine fluid, significant differences were limited to the metestrus and dioestrus phases, which might partially result from experimental variations (Siregar et al. 2019).

Beta Glbs (1 and 2) also showed stage‐specific differences. In serum, beta‐Glb levels were higher in the 16% protein group across most phases except metestrus, where the 19% group showed elevated levels. However, there were no significant differences in uterine fluid beta Glb levels between the groups. These patterns may reflect postpartum metabolic changes, favouring the 16% protein diet.

Gamma Glb concentrations in serum were significantly higher in the 16% group during proestrus, oestrus and dioestrus, possibly due to compensatory responses to reduced protein intake. This is consistent with research showing increased gamma‐Glb levels during dietary deficiency. The observed decrease in Alb in the 16% group may also contribute to a relative increase in gamma Glb levels, as indicated by changes in the Alb‐to‐Glb ratio.

In conclusion, dietary protein levels significantly affect serum and uterine protein profiles during the oestrous cycle, with clear implications for reproductive physiology in dairy cows. These findings underscore the need for balanced protein and energy nutrition to support optimal reproductive performance and metabolic health.

### 4.2 Correlation of Protein and Reproductive Factors Investigated

The dynamic changes in protein correlations throughout the reproductive cycle suggest stage‐specific modulation of the uterine environment. In proestrus, the significant negative correlation of alpha Glb 2 may indicate its regulatory influence during the preparation phase of the reproductive cycle. The generally weaker correlations in this stage imply limited protein interaction. Oestrus showed marked shifts with strong correlations involving beta Glb 1 and gamma Glb, reflecting their potential roles in ovulation and uterine receptivity. These changes highlight the importance of protein dynamics in facilitating reproductive events.

Metestrus presented a complex pattern with both positive and negative correlations, suggesting intricate protein interactions during early luteal development. During dioestrus, the exceptionally strong positive correlation of alpha Glb 2 alongside the strong negative correlation of gamma Glb implies opposing functions in maintaining luteal phase stability. Beta Glb 1's moderate positive correlation further supports its involvement in this phase. Overall, alpha Glb 2 and gamma Glb appear to be key modulators, exhibiting opposing influences that may be critical for the regulation of the uterine environment throughout the reproductive cycle.

From a practical standpoint, this study supports the recommendation that dietary protein in high‐producing dairy cows should be carefully optimized. Balancing rumen‐degradable and non‐degradable protein fractions is essential to avoid nitrogen overload and maintain a reproductive environment conducive to fertility.

Future research should explore a broader range of dietary protein levels, including values below 14% and above 20%, to better understand the thresholds at which protein intake begins to negatively or positively influence reproductive physiology. In addition, the specific role of protein quality—particularly the ratio of rumen‐degradable to non‐degradable protein—should be examined to clarify its effect on metabolic and hormonal pathways involved in fertility. Integrating hormonal profiling and gene expression analysis related to ovarian and uterine function could provide deeper insight into the molecular mechanisms by which dietary protein modulates reproductive performance. Long‐term studies assessing key fertility outcomes such as calving intervals, embryo viability and early embryonic loss are also necessary to translate biochemical findings into practical, herd‐level recommendations for improving reproductive efficiency in dairy cows.

## 5 Conclusion

This study demonstrated that increasing dietary protein from 16% to 19% significantly altered the protein composition of uterine fluid—specifically TP, Alb, alpha Glb and gamma Glb—during key phases of the oestrous cycle. However, changes in blood serum proteins were less consistent, and higher protein intake did not translate into improved fertility outcomes. These findings suggest that excess dietary protein may disrupt the reproductive environment, particularly within the uterus, potentially impairing fertility. From a practical standpoint, optimizing—not maximizing—dietary protein intake is crucial for supporting reproductive health in dairy cows. Future studies should investigate a wider range of protein levels and assess long‐term reproductive outcomes to develop precise nutritional strategies that enhance fertility without compromising production.

## Author Contributions

**Akbar Pirestani**: resources, funding acquisition, conceptualization, methodology, supervision, validation, project administration, writing – review and editing. **Elmira Ziya Motalebipour**: data curation, investigation, formal analysis, visualization, writing – original draft.

## Ethics Statement

The authors have nothing to report.

## Conflicts of Interest

The authors declare no conflicts of interest.

## Peer Review

The peer review history for this article is available at https://www.webofscience.com/api/gateway/wos/peer‐review/10.1002/vms3.70631.

## Data Availability

The data presented in this study are available upon request from the corresponding author.
